# Oncogenetics training in Brazilian medical genetics residency programs: current landscape and challenges

**DOI:** 10.1007/s12687-026-00916-5

**Published:** 2026-06-29

**Authors:** Amaro Freire de Queiroz Júnior, Angelina Xavier Acosta, Carlos Eduardo Steiner, Cezar Antônio Abreu de Souza, Chong Ae Kim, Fernando Regla Vargas, Maria Denise Fernandes Carvalho de Andrade, Patrícia Santana Correia, Paulo Ricardo Gazzola Zen, Romina Soledad Heredia, Victor Evangelista de Faria Ferraz, Débora Gusmão Melo, Patrícia Ashton-Prolla

**Affiliations:** 1 Instituto Nacional de Ciência e Tecnologia de Doenças Raras (InRaras) - Hospital de Clínicas de Porto Alegre (HCPA), Porto Alegre, Brazil; 2 Programa de Residência Médica em Genética Médica do Complexo Hospital Universitário Professor Edgar Santos/Maternidade Climério de Oliveira/Universidade Federal da Bahia (Hupes-UFBA), Salvador, Brazil; 3 Programa de Residência Médica em Genética Médica da Universidade Estadual de Campinas (UNICAMP), Campinas, Brazil; 4 Programa de Residência Médica em Genética Médica do Hospital das Clínicas da Universidade Federal de Minas Gerais (UFMG), Belo Horizonte, Brazil; 5 Programa de Residência Médica em Genética Médica do Hospital das Clínicas da Faculdade de Medicina da Universidade de São Paulo (FMUSP), São Paulo, Brazil; 6 Programa de Residência Médica em Genética Médica do Hospital Universitário Gaffrée e Guinle/Universidade Federal do Estado do Rio de Janeiro (UNIRIO) e Instituto Oswaldo Cruz, Fundação Oswaldo Cruz (FIOCRUZ), Rio de Janeiro, Brazil; 7 Programa de Residência Médica em Genética Médica do Hospital Universitário do Ceará/Escola de Saúde Pública do Ceará (ESP/CE), Fortaleza, Brazil; 8 Programa de Residência Médica em Genética Médica do Instituto Nacional de Saúde da Mulher, da Criança e do Adolescente Fernandes Figueira (IFF/Fiocruz), Rio de Janeiro, Brazil; 9 Programa de Residência Médica em Genética Médica da Universidade Federal de Ciências da Saúde de Porto Alegre (UFCSPA)/Irmandade da Santa Casa de Misericórdia de Porto Alegre (ISCMPA), Porto Alegre, Brazil; 10 Programa de Residência Médica em Genética Médica da Escola Superior de Ciências da Saúde (ESCS), Brasília, DF Brazil; 11 Programa de Residência Médica em Genética Médica do Hospital das Clínicas da Faculdade de Medicina da Universidade de São Paulo (HCFMRP-USP), Ribeirão preto, Brazil; 12 Departamento de Genética da Faculdade de Medicina de Ribeirão Preto da Universidade de São Paulo, Ribeirão Preto, Brazil; 13 Programa de Residência Médica em Genética Médica da Escola Paulista de Medicina, Universidade Federal de São Paulo (UNIFESP), São Paulo, Brazil; 14 Programa de Residência Médica em Genética Médica do Serviço de Genética Médica, Hospital de Clínicas de Porto Alegre (HCPA), Porto Alegre, Brazil; 15 Departamento de Genética, Universidade Federal do Rio Grande do Sul, Porto Alegre, Brazil; 16 Instituto Nacional de Ciência e Tecnologia de Prevenção e Saúde de Precisão em Oncogenética (Prev-Onco), Porto Alegre, Brazil

**Keywords:** Medical residency, Medical genetics, Graduate medical education, Oncogenetics, Hereditary cancer syndromes, Brazil

## Abstract

Oncogenetics focuses on identifying and managing hereditary cancer predisposition, enabling risk-reducing interventions and targeted therapies. This descriptive study evaluated the landscape of oncogenetics training within Medical Genetics Residency Programs in Brazil. Data were collected through online questionnaires and interviews with program supervisors. We assessed rotation availability, timing and duration, clinical and theoretical workload, multidisciplinary care, and competency assessment. Data were analyzed using descriptive statistics. All twelve accredited programs participated. Eleven (92%) offered oncogenetics training, primarily in the third year of residency. While half of the programs provided internal rotations, all allowed external rotations to compensate for local infrastructure limitations. Residents completed a mean of 250.1 practical hours (SD 126.5; range 40–400) over approximately 6.5 months, seeing about 13 patients per week (SD 9.2; range 5–30). Theoretical training averaged 31.3 h (SD 26.8; range 4–70), resulting in a mean total training workload of 258.7 h (SD 159.1; range 4–470). Multidisciplinary care and research opportunities were common; however, formal competency assessments specific to oncogenetics were infrequent. Considerable variability in workload and clinical settings was observed across programs. The findings indicate that oncogenetics is well integrated into Brazilian medical genetics training, aligning with the growing importance of genomic medicine in oncology. Nevertheless, the marked heterogeneity underscores the need for standardized training approaches. The study suggests that establishing inter-institutional networks and formalizing external rotations may help reduce geographic and technological disparities and support more consistent oncogenetics training in medical genetics residency programs across Brazil.

**Supplementary Information:**

The online version contains supplementary material available at https://doi.org/10.1007/s12687-026-00916-5.

## Introduction

Medical Genetics is an increasingly important specialty in contemporary medicine, dedicated to the diagnosis, management, and counseling of individuals and families affected by genetic conditions (Curd et al. 2025). In Brazil, since its official recognition by the Federal Council of Medicine (in Portuguese: Conselho Federal de Medicina, CFM) in 1983, the specialty has progressively expanded through training programs, clinical services, and a growing professional workforce (CFM 1983; Melo et al. 2017). A key component of this expansion is the training of new specialists through Medical Genetics Residency Programs (MGRPs). Currently, Brazil has 14 MGRPs accredited by the National Commission for Medical Residency (in Portuguese: Comissão Nacional de Residência Médica, CNRM), offering 32 residency positions in 2026—an increase from 12 programs and 29 positions in 2025. These programs are unevenly distributed across four of the five Brazilian regions: seven in the Southeast, three in the Northeast, two in the Central-West, and two in the South (SBGM 2025). The strengthening of the specialty has been further driven by the National Policy for Comprehensive Care for People with Rare Diseases (Brasil 2014), which currently includes approximately 50 healthcare centers accredited by the Ministry of Health (Brasil 2025). This expansion is reflected in a 174% increase in the number of medical geneticists in the country between 2011 and 2024 (Horovitz et al. 2024; Scheffer 2025).

Despite these advances, significant challenges remain, including regional service concentration, laboratory shortages for genetic testing, and insufficient integration with primary care (Bonilla et al. 2022; de Oliveira and Lopes-Cendes 2025). In addition, the number of specialists is still insufficient to meet national demand (de Lima et al. 2025), an issue also reported in other regions, including Latin America, Europe, the United States, and Australia (Dragojlovic et al. 2020).

Within this scenario, oncogenetics emerges as a strategic area of practice, as at least 10% of cancer patients are estimated to have a hereditary predisposition (in Brazil, approximately 70,000 new cases annually) (INCA 2026; Leite et al. 2022; de Oliveira et al. 2022). These patients often require specialized management due to early onset of disease and the increased risk for developing multiple tumors. Consequently, the rapid expansion of precision medicine has intensified the need for adequate professional training, particularly given the documented gaps in genetics education among healthcare professionals (Houwink et al. 2015; Stellacci et al. 2024).

In Brazil, this relevance was further reinforced in 2024 when Oncogenetics was officially recognized as an area of medical practice by the Brazilian Medical Association (in Portuguese: Associação Médica Brasileira, AMB) and CFM (CFM 2024). This recognition resulted from the joint efforts of five medical societies, including the Brazilian Society of Medical Genetics and Genomics (in Portuguese: Sociedade Brasileira de Genética Médica e Genômica, SBGM). However, important inequalities exist between the Brazilian public and private healthcare systems regarding the management of oncologic patients, particularly the limited availability of genetic testing within the public health system, in contrast to the mandatory coverage in the private health system since 2014. In this context, the development of specialized competencies depends not only on individual learning but also on the educational environments where residents are trained. From the perspective of the “Communities of Practice” framework (Li et al. 2009; Ranmuthugala et al. 2011), professional learning occurs through participation in clinical activities and interactions with experienced practitioners across different clinical environments.

International literature highlights that formal education in cancer genetics—including genomic data management and precision therapeutics—is increasingly recognized as essential. Professional societies such as the American Society of Clinical Oncology (ASCO) have emphasized the need for structured training in this field (ASCO 1997; Robson et al. 2015). Nevertheless, studies demonstrate that while structured oncogenetics training improves consultation skills, oncogenetic education in general remains suboptimal and requires continuous revision (Houwink et al. 2014a, b, 2015).

However, little is known about how such training is organized within Brazilian MGRPs. There is a lack of systematic information regarding the educational activities, clinical exposure, and institutional resources available to residents in this field. Examining how these programs organize oncogenetics training is essential to ensure that future specialists are adequately prepared to meet the growing demands of precision oncology. From a community genetics perspective, the training of professionals capable of providing hereditary cancer risk assessment and genetic counseling is particularly relevant, as workforce preparation directly influences the organization, accessibility, and quality of genetic services within healthcare systems. Therefore, this study aims to describe the characteristics of oncogenetics training offered by the 12 Medical Genetics Residency Programs in Brazil in 2025 and to identify strengths and opportunities for improvement in oncogenetics workforce training.

## Methodology

### Study design and data collection

A cross-sectional descriptive study was conducted to characterize the structure and training practices related to oncogenetics in Medical Genetics Residency Programs in Brazil. To our knowledge, this study provides the first systematic national overview of oncogenetics training in these programs.

Data collection took place in two complementary stages. The first stage was conducted in October 2024; at that time, a structured online questionnaire consisting of 25 items was administered to the supervisors of the 12 active MGRPs in Brazil. The questionnaire explored key structural and educational components of oncogenetics training, including: availability of an oncogenetics rotation; hours of hands-on clinical activities; year of residency in which training occurs; duration of training activities; number of patients attended; theoretical and practical curricular content; competency assessment; multidisciplinary collaboration; type of supervision; participation in research and outreach activities; institutional partnerships; and affiliation with the Brazilian Hereditary Cancer Network (ReBraCH). The complete questionnaire is provided as Supplementary Material.

The second stage was conducted between March and May 2025, during which individual remote interviews were conducted with one representative from each residency program, either the program supervisor or a designated preceptor indicated by the supervisor. All interviews were conducted by a single researcher (P.A.P.), thereby helping to ensure consistency in data collection. The semi-structured interviews followed the same questionnaire used in the online survey and were intended exclusively to confirm responses, clarify uncertainties, and obtain additional details, particularly regarding curricular content and teaching strategies. This two-stage approach allowed the verification of questionnaire responses and provided additional contextual information regarding the organization of oncogenetics training across programs.

### Participants

The participants in this study were the supervisors or designated preceptors of the MGRPs included in the survey. Contact information for the program supervisors was provided by the Brazilian Society of Medical Genetics and Genomics (SBGM). All 12 MGRPs accredited by the National Commission for Medical Residency at the time of data collection were invited to participate. All invited programs agreed to participate in the study. Questionnaire responses and follow-up interviews were obtained from one representative of each of the 12 residency programs. Participants were either program supervisors or preceptors designated by the supervisors and, in all cases, were directly involved in residency training activities within their respective programs.

In addition to providing study data, these participants were included as co-authors and contributed to the analysis and critical discussion of the results, helping to ensure the accuracy and contextual interpretation of the information reported for each program.

Because the study collected institutional information about residency training structures rather than personal or sensitive data, and because all participants voluntarily contributed to the study and were directly involved in the analysis and interpretation of the results, formal ethical review was not required under institutional guidelines.

### Data analysis

Data were analyzed descriptively using measures of central tendency and dispersion for quantitative variables and frequency distributions for categorical variables. Information obtained the follow-up interviews was not subjected to formal qualitative analysis. Instead, interview data were used exclusively to verify questionnaire responses, resolve inconsistencies or ambiguities, and provide complementary contextual information to support the descriptive characterization of oncogenetics training practices across programs.

## Results

All 12 MGRPs accredited by the National Commission for Medical Residency in Brazil as of 2025 participated in the survey, resulting in a 100% response rate. Most programs were located in the Southeast region (7/12; 58%), followed by programs in the South, Northeast, and Central-West regions. Nine out of the 12 genetics services hosting MGRPs were accredited as Reference Services under the National Policy for Comprehensive Care for People with Rare Diseases within the Brazilian Unified Health System (in Portuguese: Sistema Único de Saúde, SUS). As shown in Table 1, the residency programs were established between 1977 and 2024, reflecting the progressive expansion of medical genetics training in Brazil over the past five decades. Programs offered between one and five residency positions per year, with most programs offering two positions annually. The number of core preceptors ranged from three to twelve physicians across institutions.

**Table 1:** Characteristics of Medical Genetics Residency Programs (MGRPs) in Brazil in 2025

Year of initiation	Region of the country	Number of positions offered per year	Number of core residency preceptors *
1977	Southeast	3	7
1988	Southeast	2	4
1989	South	3	11
1994	South	1	3
2004	Southeast	2	7
2005	Southeast	2	6
2005	Southeast	2	8
2007	Center-West	2	4
2009	Southeast	5	5
2012	Southeast	2	5
2013	Northeast	3	12
2024	Northeast	2	9

*Core team preceptors only; excludes staff from occasional rotations outside the medical genetics service (e.g., one-month rotations) within the institution

Table 2 summarizes the institutional characteristics, clinical workload, and educational components of oncogenetics training across programs. Among the 12 programs, 11 (92%) routinely offered an oncogenetics rotation. Of these programs, five provided training entirely within their own institution, one combined activities between the home institution and partner centers, with the latter being responsible for the greater workload and patient volume, and five did not have their own oncogenetics clinical service and relied entirely on partner institutions in the public, private, or philanthropic sectors. Eight of the 11 MGRPs had preceptors affiliated with the Brazilian Hereditary Cancer Network (in Portuguese: Rede Brasileira de Cancer Hereditario, Rebrach).

**Table 2:** Educational structure and clinical workload of oncogenetics training across Brazilian Medical Genetics Residency Programs (MGRPs)

Educational components of oncogenetics training	Medical Genetics Residency Programs											
	1	2	3	4	5	6	7	8	9	10	11	12
Offers oncogenetics rotation	Yes	Yes	Yes	Yes	No	Yes	Yes	Yes	Yes	Yes	Yes	Yes
Rotation conducted within the home institution	Yes	Yes	Yes	Yes	NA	No	No	No	No	^**†**^ Yes	No	Yes
Formal partnership with other institutions for oncogenetics rotation	No	Yes	Yes	No	No	Yes	Yes	No	Yes	Yes	Yes	No
Hours of practical hands-on activities	400	258	400	360	NA	96	144	40	320	352	160	230
Average consultations per week	6	6	6	16	NA	5	15	8	18	28	30	5
Hours of theoretical activities (lectures and meetings)	60	12	70	70	4	0	48	12	16	8	4	40
Total hours of oncogenetics training	460	270	470	430	4	96	192	52	336	360	164	270
Duration of activities (months)	10	12	14	5	NA	6	3	2	2	4	1	13
Year of residency of oncogenetics activities	R1, R2, R3	R1, R2, R3	R2, R3	R3	NA	R3	R3	R2, R3	R2, R3	R3	R3	R2, R3
Trainee evaluation specifically in oncogenetics	No	Yes	No	No	No	No	No	No	No	No	Yes	No
Research activities in oncogenetics	Yes	Yes	Yes	Yes	No	No	No	Yes	No	No	Yes	Yes
Allows oncogenetics rotations in other institutions	Yes	Yes	Yes	Yes	Yes	Yes	Yes	Yes	Yes	Yes	Yes	Yes
Clinical activities are in multidisciplinary environment	No	Yes	No	Yes	No	No	Yes	Yes	Yes	Yes	No	Yes
Professionals involved in the oncogenetics clinic	NA	PedOnc; Bio	NA	Nurse; Onco	NA	NA	Psy	Psy; BreastSurg	Surg; Nurse	Psy; Nurse; Onco	NA	Nurse; Onco

Considering the 11 MGRPs that offered an oncogenetics rotation, the mean number of hours of practical hands-on activities was 250.1 (40–400; SD ± 126.5), distributed over a mean duration of 6.5 months (1–14 months; SD ± 4.8). The mean number of consultations per resident per week was 13 (5–30 patients; SD ± 9.2). Two MGRPs distributed these activities across all three years of residency (R1, R2, and R3); four conducted them during the second and third years (R2 and R3); and five included activities only in the third year (R3).

Regarding theoretical training, one of the 12 MGRPs did not have a specific structured teaching program in oncogenetics. Among the remaining programs, the mean number of hours dedicated to theoretical training was 31.3 (4–70 h; SD ± 26.8). Interestingly, the only program that did not offer an oncogenetics rotation included 4 h of theoretical activities in oncogenetics. The mean total training workload in oncogenetics (practical and theoretical) across the 12 MGRPs was 258.7 h (4–470; SD ± 159.1).

In seven of the 11 MGRPs offering oncogenetics training (64%), there was a multidisciplinary approach, reflecting the participation of professionals from different healthcare fields in oncogenetics care. Participation of psychologists, clinical oncologists, breast surgeons, surgical oncologists, nurses, and biologists was reported, illustrating the incorporation of multidisciplinary perspectives into the clinical management of patients with suspected or confirmed hereditary cancer.

All programs reported conducting institutional evaluations of resident performance; however, only two programs included a specific theoretical assessment focused on oncogenetics. Finally, seven MGRPs offered opportunities for residents to participate in oncogenetics research activities. In addition, all 12 programs allowed external oncogenetics rotations of up to one month, selected by the residents, enabling exposure to diverse institutional settings and clinical practices.

## Discussion

This study investigated the characteristics, structure, and workload of oncogenetics training across all 12 MGRPs accredited in Brazil up to 2025. This landscape should be considered dynamic and subject to change, requiring periodic updates as residency programs, genomic technologies, and healthcare demands evolve. Although oncogenetics has been integrated into medical genetics for decades, systematized data on the implementation and outcomes of educational practices remain scarce globally (Korf 2014; Moog et al. 2025). Consequently, understanding how oncogenetics training is structured within residency programs represents an important step toward evaluating whether current educational models are aligned with the growing demand for hereditary cancer risk assessment. This issue is particularly relevant for community genetics, as the availability of adequately trained professionals directly affects the capacity of healthcare systems to identify families at risk and deliver equitable genetic services.

Our findings reflect a global trend: the growing need to strengthen genetic literacy as genomic information becomes increasingly central to cancer management. Educational interventions have been shown to improve genetic consultation skills and clinical management, although baseline knowledge in oncogenetics often remains limited (Houwink et al. 2014a, b, 2015; Murray et al. 2021; Marzuillo et al. 2014; Panic et al. 2014; Stellacci et al. 2024). The demand for structured training reflects a persistent global mismatch between the rapid evolution of precision oncology and the pace of medical education (Tutika et al. 2023; Park et al. 2024). A study conducted with oncologists in the United Kingdom revealed that 92.7% identified a need for more training in genomics and 80.7% reported a lack of training in genetic counseling, despite prescribing germline tests (Tutika et al. 2023). This finding illustrates how clinical practice and precision oncology are evolving more rapidly than structured medical education. This gap provides an important context for understanding the current state of oncogenetics training in Brazil, where its inclusion in nearly all curricula is promising, yet significant variability exists among programs. Theoretical workload ranged from 0 to 70 h across programs, and supervised clinical exposure varied from 40 to 400 h, highlighting the absence of standardized training environments in a field that represents a growing component of clinical genetics practice.

Similar concerns have been raised internationally. In the United States, the ASCO Cancer Genetics Curriculum and subsequent policy statements have defined hereditary cancer risk assessment, family history collection, genetic test selection and interpretation, communication skills, and long-term management of high-risk individuals as core competencies for oncology practice (ASCO 1997; Robson et al. 2015). Likewise, the ESMO/ASCO Global Curriculum in Medical Oncology includes competencies related to cancer susceptibility syndromes, germline testing, and referral for genetic counseling within modern oncology training (Cufer & Kosty 2023). Studies from Europe and Australia have likewise emphasized that effective oncogenetics education requires structured training pathways and competency frameworks that extend beyond theoretical knowledge to include clinical decision-making, communication, and multidisciplinary practice (Park et al. 2024). Viewed in this international context, the incorporation of oncogenetics into nearly all Brazilian Medical Genetics Residency Programs represents an important achievement; however, the substantial variability in workload and educational experiences suggests that, unlike several contemporary international frameworks that specify core competencies and expected areas of proficiency, Brazilian residency training still relies largely on local curricular arrangements, resulting in heterogeneous educational pathways and potentially variable competency acquisition across programs. In contrast to competency frameworks that increasingly advocate longitudinal and supervised exposure to hereditary cancer care, practical training experiences in Brazil remain highly dependent on local institutional resources and partnerships. This variability likely reflects, at least in part, the absence of nationally standardized requirements for oncogenetics training and assessment. Understanding the regulatory factors that shape residency training is therefore essential to explain why this heterogeneity persists.

One possible explanation for this lack of standardization lies in the differing regulatory frameworks guiding residency training. While oncogenetics is included in the competency frameworks of both Medical Genetics and Clinical Oncology residency programs, only Clinical Oncology programs currently specify a dedicated workload for this topic. These programs recommend that 10% of the final two years of training (R2 and R3) be dedicated to oncogenetics, corresponding to approximately 576 h of training (Brasil 2019a). In contrast, the 2019 competency framework for Medical Genetics Residency Programs recognizes oncogenetics as a training component but does not assign a specific workload (Brasil 2019b). To address this gap, the Brazilian Society of Medical Genetics and Genomics (SBGM) held technical workshops in 2025 with residency supervisors and recent graduates to update the framework. The proposed revisions were submitted to the National Commission for Medical Residency but have yet to undergo institutional review or receive regulatory approval. Consequently, the 2025 document currently serves a prospective and advisory role, aiding training discussions without replacing the officially approved framework. Despite its non-regulatory status, its emphasis on genomic interpretation, therapeutic decision-making, and communication of molecular information brings the Brazilian discussion closer to contemporary international competency frameworks in hereditary cancer genetics and precision oncology.

A comparison between the 2019 and 2025 frameworks reflects an evolution in the conceptual approach related to oncogenetics training (Table 3). While both frameworks introduce theoretical foundations in the second year and concentrate advanced content in the third year of residency, the 2025 proposal reflects the technological and conceptual expansion of the field. In this updated model, cancer-related competencies move from general mentions of personalized medicine into a new framework that explicitly integrates germline and somatic genomics, variant interpretation, and clinical communication of molecular results. This transition redefines the medical geneticist’s scope: the role now extends beyond clinical diagnosis to include the interpretation of large-scale genomic and epigenomic datasets and active engagement with therapeutic approaches. Notably, genetic counseling is elevated to a program-wide general objective, shifting the educational focus from memorizing phenotypic patterns toward developing advanced cognitive skills in clinical bioinformatics and ethical decision-making regarding variants of uncertain significance.

**Table 3:** Comparative evolution of oncogenetics competencies between the 2019 National Commission of Medical Residency (CNRM) framework and the 2025 Brazilian Society of Medical Genetics and Genomics (SBGM) proposal

Theme	2019 Competency Framework (current)*	2025 Competency Framework (proposed)**
Knowledge Management	Focuses on the “Genetic basis of cancer” (R2) and “Hereditary cancer syndromes” (R3). Mentions “Personalized Medicine” as a broad topic in R3.	Maintains the focus on the “Genetic basis of cancer” in R2. Expands R3 content to include “Hereditary cancer predisposition and its main syndromes” and the “Integration of somatic and germline data in the investigation of hereditary cancer.” “Personalized medicine” remains in R3.
Clinical Assessment & Management	Focuses on developing diagnostic hypotheses and investigation plans based on clinical data. Emphasis is placed on clinical management of oncogenetic diseases in R3.	Maintains the emphasis in R3. Expands in R3 to require the integration of clinical and laboratory (molecular) data for diagnosis and engagement with multidisciplinary therapeutic approaches.
Diagnostic & Genomic Tools	Indirectly addresses molecular tools through the mastery of databases for variant interpretation in R3.	Robustly expands in R3 to include critical analysis of genomic and epigenomic data, alongside advanced clinical bioinformatics for variant interpretation.
Genetic Counseling	Defined as a specific R3 skill focused on guiding families about the relevance, limitations, and ethics of predictive testing.	Elevated to a General Objective of the residency program. Requires mastery of all counseling stages, including risk calculation and complex molecular result communication.

* The 2019 framework is the current legally approved document in Brazil

** The 2025 framework is a proposal developed by the SBGM currently under advisory status and awaiting institutional approval by regulatory authorities

In addition to curricular differences, our findings also reveal important variation in the organization of practical training. The mean workload of practical activities (250.1 h) indicates that substantial supervised clinical exposure is available in several programs, representing a positive aspect of current residency training. However, only half of the MGRPs offer these activities within their own institutions, and many residents complete their oncogenetics training in partner institutions. This situation may affect training quality by limiting pedagogical continuity, reducing opportunities for standardized supervision, and potentially fragmenting the clinical learning experience. Furthermore, the model of immersion—whether as a concentrated rotation or a longitudinal activity—also influences the depth of clinical experience.

The practice setting itself also contributes to this variability. Programs with internal oncogenetics services generally provide training in outpatient clinics linked to the Brazilian public healthcare system (SUS), whereas rotations conducted at partner institutions frequently occur in private healthcare settings. Access to genetic testing, molecular diagnostics, and targeted therapies differs substantially between these systems. In the public healthcare system, genetic testing in oncogenetics remains limited and often restricted to research settings, whereas private institutions may provide broader access to genomic technologies (Horovitz et al. 2024). As a result, residents may experience unequal exposure to essential diagnostic and therapeutic tools for oncogenetics practice.

These structural differences in practice settings carry important educational implications for how residents experience oncogenetics training. According to Situated Learning Theory, professional competence develops through “legitimate peripheral participation” within communities of practice (O’Brien and Battista 2020). This framework helps explain the institutional variations in oncogenetics training identified in our study. In programs lacking internal oncogenetics services, residents may face challenges integrating into stable communities of practice due to inconsistent supervision. In the Brazilian context, our data indicate that while nearly all programs provide oncogenetics training, the quality and continuity of this engagement can differ. Additionally, the contrast between the public health system (SUS), where testing is often restricted to research settings, and the private sector, which generally offers broader genomic access, may contribute to a “hidden curriculum” (Zanolli et al. 2020). In this context, limited resources might be interpreted as inherent limits of the specialty itself, rather than as external structural constraints (Basu et al. 2012; Benamer et al. 2023).

In our study, only two programs reported having a specific theoretical assessment in oncogenetics. The absence of standardized assessment strategies in most programs suggests that training often follows a time-based rather than a competency-based medical education (CBME) model (Khachadoorian-Elia 2025). Adopting CBME principles could ensure that essential professional activities are achieved regardless of the institutional setting (Cooper and Holmboe 2025; Schumacher et al. 2020). In this context, the implementation of Entrustable Professional Activities (EPAs) acts as a critical tool to translate broad competencies into observable clinical work, enabling faculty to identify skill gaps and align educational outcomes with patient safety (Bramley and McKenna 2021).

These findings are also consistent with international recommendations that increasingly advocate competency-based approaches to cancer genetics education. The ASCO curriculum and more recent educational frameworks emphasize that competency assessment should extend beyond attendance or exposure time and include demonstrable abilities in pedigree construction, hereditary cancer risk assessment, test interpretation, counseling regarding the benefits and limitations of testing, and formulation of surveillance and risk-reduction recommendations (ASCO 1997; Robson et al. 2015; Park et al. 2024). From this perspective, the limited use of formal assessment strategies observed in Brazilian programs may represent an important opportunity for future curriculum development. The adoption of nationally defined competencies and assessment tools could help ensure more homogeneous training outcomes despite differences in institutional resources and clinical settings. Such an approach would also facilitate benchmarking across programs and enable future evaluations of whether residency training effectively prepares medical geneticists for the expanding demands of hereditary cancer care and precision oncology.

The reliance on external training sites identified in our study, rather than representing solely a limitation, may also create opportunities to leverage inter-institutional networks, virtual tumor boards, and rotation-sharing models to address geographic and technological disparities. These structured collaborations may allow residents to bridge gaps between high-volume clinical services and specialized centers equipped with advanced genomic and bioinformatics infrastructure (Weitzel et al. 2011). In this context, “away rotations” become crucial when a primary institution lacks sufficient case volume to fulfill minimum training requirements, especially in specialized or regionally focused fields (Gephart et al. 2015). Educational collaboratives are widely recognized as an effective means for programs to meet both educational and community goals (Ormsby et al. 2025). Evidence from multi-institutional partnerships, such as those among pediatric residency programs in California, shows that these networks reduce faculty isolation, uncover curricular gaps, and help overcome resource constraints (Chamberlain et al. 2013). To effectively deliver state-of-the-art oncology and genetics training, multimodal strategies that integrate distance learning with in-person experience and ongoing practice-based support have proven most successful (Weitzel et al. 2011).

Finally, training must encompass the clinical management of hereditary cancer risk. While antineoplastic prescription is reserved for oncologists (Brasil 2004), geneticists are crucial in multidisciplinary decision-making. Integration with teams in surgical oncology, psychology, and patient navigation is essential for patient-centered care (Aissaoui et al. 2014; Grant et al. 2025). In our study, seven programs reported active multidisciplinary engagement, a known predictor of the development of an expanded professional identity through workplace-based interprofessional learning (Shinkaruk et al. 2023). Strengthening these collaborative environments is vital to bridge the gap between theoretical knowledge and the demands of precision oncology. Taken together, these findings suggest that improving the structure and coordination of oncogenetics training may have implications beyond residency education. From a broader community genetics perspective, strengthening oncogenetics training may help expand both the availability and the quality of hereditary cancer risk assessment services, particularly in healthcare systems where access to genetic expertise remains limited.

Although the participation of all accredited programs ensured national coverage, this study has several limitations. First, the data were based on self-reported information provided by program supervisors or designated preceptors, which may introduce social desirability bias and might not fully capture variations in day-to-day training practices. Second, the study focused primarily on structural and curricular aspects of oncogenetics training and did not directly assess educational outcomes or the actual competencies achieved by residents. Future studies should also examine whether differences in curricular structure and assessment practices translate into measurable differences in competency acquisition and preparedness for independent oncogenetics practice. Third, although the questionnaire and interviews sought to standardize data collection, differences in institutional organization and interpretation of training activities may have influenced how certain aspects of the programs were reported. Finally, the study reflects the perspectives of educators; future research incorporating the experiences of residents and recent graduates will be important to more comprehensively evaluate the effectiveness of oncogenetics training in Brazil.

## Conclusions

This study provides the first national-level overview of oncogenetics training across Medical Genetics Residency Programs in Brazil. Although oncogenetics is incorporated into nearly all curricula, substantial variability exists in workload, training environments, and assessment strategies. These findings highlight both existing strengths in residency training and important opportunities to improve the preparation of the oncogenetics workforce.

These findings highlight the importance of developing a more structured national curriculum framework that incorporates competencies related to therapeutic management and precision oncology, enabling medical geneticists to act as key collaborators within multidisciplinary teams. Drawing on emerging international frameworks in hereditary cancer genetics, future curricular reforms should also establish explicit competencies and standardized assessment strategies that promote more consistent educational outcomes across residency programs. The establishment of inter-institutional networks and national communities of practice may represent an important mechanism for implementing these changes by facilitating the sharing of best practices, digital educational resources, and educational assessment tools, thereby helping to reduce current disparities among programs and healthcare systems.

In addition, residency programs should ensure consistent exposure to multidisciplinary clinical environments in which geneticists collaborate with surgical oncology, clinical oncology, and patient navigation teams. By strengthening collaborative educational strategies and implementing competency-based training frameworks, Brazil can better prepare future medical geneticists to contribute not only to the diagnosis of hereditary cancer syndromes but also to hereditary cancer risk assessment, genomic-informed clinical management, and cancer prevention within the expanding field of precision oncology.

## Supplementary Information

Below is the link to the electronic supplementary material.


Supplementary Material 1


## Data Availability

All relevant data are within the paper. Should anything else be needed, the corresponding author can be directly contacted at pprolla@hcpa.edu.br.

## References

[CR1] Aissaoui S, Aissaoui H, Giraud S, Pinson S, Calender A (2014) Development of multidisciplinary committees for decision making and care management in hereditary colon cancer: the French state of the art. J Community Genet 5:185–189. 10.1007/s12687-013-0167-8 24018618 PMC3955458

[CR2] ASCO, American Society of Clinical Oncology (1997) Resource document for curriculum development in cancer genetics education. J Clin Oncol 15:2157–2169. 10.1200/JCO.1997.15.5.2157 9164231

[CR3] Basu S, Andrews J, Kishore S, Panjabi R, Stuckler D (2012) Comparative performance of private and public healthcare systems in low- and middle-income countries: a systematic review. PLoS Med 9:e1001244. 10.1371/journal.pmed.1001244 22723748 PMC3378609

[CR4] Benamer HT, Alsuwaidi L, Khan N et al (2023) Clinical learning environments across two different healthcare settings using the undergraduate clinical education environment measure. BMC Med Educ 23:495. 10.1186/s12909-023-04467-y 37407987 PMC10324227

[CR5] Bonilla C, Albuquerque Sortica V, Schuler-Faccini L, Matijasevich A, Scheffer MC (2022) Medical geneticists, genetic diseases and services in Brazil in the age of personalized medicine. Pers Med 19:549–563. 10.2217/pme-2021-0153 36317557

[CR6] Bramley AL, McKenna L (2021) Entrustable professional activities in entry-level health professional education: A scoping review. Med Educ 55:1011–1032. 10.1111/medu.14539 33884655

[CR11] Brasil (2004) Ministério da Saúde. Agência Nacional de Vigilância Sanitária Resolução RDC nº 220, de 21 de setembro de 2004. Aprova o Regulamento Técnico de funcionamento dos Serviços de Terapia Antineoplásica. Diário Oficial da União. https://bvsms.saude.gov.br/bvs/saudelegis/anvisa/2004/rdc0220_21_09_2004.html

[CR7] Brasil. Ministério da Educação (2019a) Resolução nº 4, de 8 de abril de 2019. Matriz de competências dos Programas de Residência Médica em Oncologia Clínica. Comissão Nacional de Residência Médica. https://portal.mec.gov.br/index.php?option=com_docman&view=download&alias=111471-04-resolucao-n-4-de-8-de-abril-de-2019-oncologia-clinica&category_slug=abril-2019-pdf&Itemid=30192

[CR8] Brasil. Ministério da Educação (2019b) Resolução nº 20, de 8 de abril de 2019. Matriz de competências dos Programas de Residência Médica em Genética Médica. Comissão Nacional de Residência Médica. https://portal.mec.gov.br/index.php?option=com_docman&view=download&alias=111641-20-resolucao-n-20-de-8-de-abril-de-2019-genetica-medica&category_slug=abril-2019-pdf&Itemid=30192

[CR10] Brasil. Ministério da Saúde (2025) Política de saúde. Secretaria de Atenção Especializada à Saúde. https://www.gov.br/saude/pt-br/composicao/saes/doencas-raras/politica-de-saude

[CR9] Brasil. Ministério da Saúde (2014) Portaria nº 199, de 30 de janeiro de 2014. Institui a Política Nacional de Atenção Integral às Pessoas com Doenças Raras. Diário Oficial da União. https://bvsms.saude.gov.br/bvs/saudelegis/gm/2014/prt0199_30_01_2014.html

[CR12] CFM, Conselho Federal de Medicina (1983) Resolução CFM nº 1.128, de 16 de setembro de 1983. Acrescenta à relação de especialidades reconhecidas a de Genética Clínica. Diário Oficial da União. https://sistemas.cfm.org.br/normas/visualizar/resolucoes/BR/1983/1128

[CR13] CFM, Conselho Federal de Medicina (2024) Resolução CFM nº 2.380, de 18 de junho de 2024. Homologa a Portaria CME nº 1/2024. Diário Oficial da União. https://sistemas.cfm.org.br/normas/visualizar/resolucoes/BR/2024/2380

[CR14] Chamberlain LJ, Wu S, Lewis G, Graff N, Javier JR, Park JS et al (2013) A multi-institutional medical educational collaborative: advocacy training in California pediatric residency programs. Acad Med 88(3):314–321. 10.1097/ACM.0b013e3182806291 23348081

[CR15] Cooper D, Holmboe ES (2025) Competency-Based Medical Education at the Front Lines of Patient Care. Reply. N Engl J Med 393:1549–1550. 10.1056/NEJMc2512926 41092340

[CR16] Cufer T, Kosty MP, Curriculum Development Subgroup—ESMO/ASCO Global Curriculum Working Group (2023) ESMO/ASCO Recommendations for a Global Curriculum in Medical Oncology Edition 2023. ESMO Open 8:101631. 10.1016/j.esmoop.2023.101631 38158226 PMC10774906

[CR17] Curd H, Gorrie A, Fennell AP (2025) From tradition to transformation: evolving models of care in clinical genetics. Curr Opin Pediatr 37:538–549. 10.1097/MOP.0000000000001502 40831355 PMC12594115

[CR18] de Lima FT, de Lima MAFD, Correia PS, Honjo RS, Maia RE, Kyosen SO, Melo DG (2025) Teaching and training of human resources for genetics and genomics in Brazil. J Community Genet 16:397–407. 10.1007/s12687-024-00726-7 39136858 PMC12321728

[CR20] de Oliveira TC, Lopes-Cendes I (2025) Population molecular genetics in Brazil: From genomic databases and research to the implementation of precision medicine. J Community Genet 16:409–420. 10.1007/s12687-024-00752-5 39557816 PMC12321696

[CR19] de Oliveira JM, Zurro NB, Coelho AVC et al (2022) The genetics of hereditary cancer risk syndromes in Brazil: a comprehensive analysis of 1682 patients. Eur J Hum Genet 30:818–823. 10.1038/s41431-022-01098-7 35534704 PMC9259741

[CR21] Dragojlovic N, Borle K, Kopac N et al (2020) The composition and capacity of the clinical genetics workforce in high-income countries: a scoping review. Genet Med 22:1437–1449. 10.1038/s41436-020-0825-2 32576987

[CR22] Gephart MH, Derstine P, Oyesiku NM, Grady MS, Burchiel K, Batjer HH et al (2015) Resident away rotations allow adaptive neurosurgical training. Neurosurgery 76(4):421–426. 10.1227/NEU.0000000000000661 25635889

[CR23] Grant AM, Taylor N, Maguire J et al (2025) A coordinated multidisciplinary model of care is needed for child and family centered care in pediatric genetic cancer risk services: a scoping review. Fam Cancer 24:55. 10.1007/s10689-025-00474-8 40540219 PMC12181217

[CR24] Horovitz DDG, Félix TM, Ferraz VEF (2024) Medical Genetics in Brazil in the 21st Century: A Thriving Specialty and Its Incorporation in Public Health Policies. Genes 15:973. 10.3390/genes15080973 39202336 PMC11353425

[CR25] Houwink EJ, Muijtjens AM, van Teeffelen SR et al (2014a) Effectiveness of oncogenetics training on general practitioners’ consultation skills: a randomized controlled trial. Genet Med 16:45–52. 10.1038/gim.2013.69 23722870 PMC3914027

[CR27] Houwink EJ, van Teeffelen SR, Muijtjens AM et al (2014b) Sustained effects of online genetics education: a randomized controlled trial on oncogenetics. Eur J Hum Genet 22:310–316. 10.1038/ejhg.2013.163 23942200 PMC3925286

[CR26] Houwink EJ, Muijtjens AM, van Teeffelen SR et al (2015) Effect of comprehensive oncogenetics training interventions for general practitioners, evaluated at multiple performance levels. PLoS ONE 10:e0122648. 10.1371/journal.pone.0122648 25837634 PMC4383330

[CR28] INCA, Instituto Nacional de Câncer (2026) Estimativa 2026: Incidência de câncer no Brasil. INCA, Rio de Janeiro. https://ninho.inca.gov.br/jspui/bitstream/123456789/17914/1/Estima2026_completo%20%281%29.pdf

[CR29] Khachadoorian-Elia HR (2025) Time-based versus competency-based medical education: Opportunities and challenges. Med Educ 59:14–16. 10.1111/medu.15567 39435953

[CR30] Korf BR (2014) The medical genetics residency milestones. J Grad Med Educ 6(1 Suppl 1):87–90. 10.4300/JGME-06-01s1-31 24701269 PMC3966601

[CR31] Leite ACR, Suzuki DA, Pereira AAL et al (2022) What can we learn from more than 1,000 Brazilian patients at risk of hereditary cancer? Front Oncol 12:963910. 10.3389/fonc.2022.963910 36132150 PMC9484549

[CR32] Li LC, Grimshaw JM, Nielsen C, Judd M, Coyte PC, Graham ID (2009) Use of communities of practice in business and health care sectors: a systematic review. Implement Sci 4:27. 10.1186/1748-5908-4-27 19445723 PMC2694761

[CR33] Marzuillo C, De Vito C, D’Addario M, Santini P, D’Andrea E, Boccia A, Villari P (2014) Are public health professionals prepared for public health genomics? A cross-sectional survey in Italy. BMC Health Serv Res 14:239. 10.1186/1472-6963-14-239 24885316 PMC4064825

[CR34] Melo DG, Germano CMR, Porciúncula CGG et al (2017) Qualification and provision of physicians in the context of the National Policy on Comprehensive Care of People with Rare Diseases in the Brazilian National Health System (SUS). Interface (Botucatu) 21:1205–1216. 10.1590/1807-57622016.0211

[CR35] Moog U, Berg J, Kerr S, Turnpenny PD, Verloes A, Working Group ETR for Medical Genetics, Zschocke J (2025) European training requirements for the specialty of medical genetics. Eur J Hum Genet 33:1121–1126. 10.1038/s41431-025-01899-6 40604116 PMC12402244

[CR36] Murray AK, McGee RB, Mostafavi RM et al (2021) Creating a cancer genomics curriculum for pediatric hematology-oncology fellows: A national needs assessment. Cancer Med 10:2026–2034. 10.1002/cam4.3787 33624449 PMC7957159

[CR37] O’Brien BC, Battista A (2020) Situated learning theory in health professions education research: a scoping review. Adv Health Sci Educ Theory Pract 25:483–509. 10.1007/s10459-019-09900-w 31230163

[CR38] Ormsby M, Shih G, Weidner A (2025) The Value of Learning Collaboratives: Experiences From Several Residency Networks. Fam Med 57(4):247–252. 10.22454/FamMed.2025.903495 40272867 PMC12147698

[CR39] Panic N, Leoncini E, Di Giannantonio P et al (2014) Survey on knowledge, attitudes, and training needs of Italian residents on genetic tests for hereditary breast and colorectal cancer. BioMed Res Int 2014:418416. 10.1155/2014/418416 25050348 PMC4094882

[CR40] Park SY, Kim Y, Katapodi MC et al (2024) Healthcare professionals’ learning needs and perspectives on essential information in genetic cancer care: a systematic review. Cancers 16:1963. 10.3390/cancers16111963 38893084 PMC11171145

[CR41] Ranmuthugala G, Plumb JJ, Cunningham FC, Georgiou A, Westbrook JI, Braithwaite J (2011) How and why are communities of practice established in the healthcare sector? A systematic review of the literature. BMC Health Serv Res 11:273. 10.1186/1472-6963-11-273 21999305 PMC3219728

[CR42] Robson ME, Bradbury AR, Arun B et al (2015) American Society of Clinical Oncology Policy Statement Update: Genetic and Genomic Testing for Cancer Susceptibility. J Clin Oncol 33:3660–3667. 10.1200/JCO.2015.63.0996 26324357

[CR43] SBGM Sociedade Brasileira de Genética Médica e Genômica (2025) Residência Médica. https://www.sbgm.org.br

[CR44] Scheffer M (2025) Demografia Médica no Brasil 2025. Ministério da Saúde. http://bvsms.saude.gov.br/bvs/publicacoes/demografia_medica_brasil_2025.pdf

[CR45] Schumacher DJ, West DC, Schwartz A et al (2020) Longitudinal Assessment of Resident Performance Using Entrustable Professional Activities. JAMA Netw Open 3:e1919316. 10.1001/jamanetworkopen.2019.19316 31940042 PMC6991321

[CR46] Shinkaruk K, Carr E, Lockyer JM, Hecker KG (2023) Exploring the development of interprofessional competence and professional identity: A Situated Learning Theory study. J Interprof Care 37:613–622. 10.1080/13561820.2022.2140129 36448594

[CR47] Stellacci E, Martinelli S, Carbone P et al (2024) Bridging the educational gaps of health professionals in oncogenomics: results from a pilot e-learning course. Front Med 11:1422163. 10.3389/fmed.2024.1422163 PMC11617149 39640978

[CR48] Tutika RK, Bennett JA, Abraham J et al (2023) Mainstreaming of genomics in oncology: a nationwide survey of the genomics training needs of UK oncologists. Clin Med (Lond) 23:9–15. 10.7861/clinmed.2022-0372 36697012 PMC11046524

[CR49] Weitzel JN, Blazer KR, MacDonald DJ, Culver JO, Offit K (2011) Genetics, genomics, and cancer risk assessment: State of the Art and Future Directions in the Era of Personalized Medicine. CA Cancer J Clin 61(5):327–359. 10.3322/caac.20128 21858794 PMC3346864

[CR50] Zanolli MB, Streit DS, Maciel DT, Muraguchi EMO, Martins MA, Tibério IFLC (2020) Differences in clerkship development between public and private Brazilian medical schools: an overview. BMC Med Educ 20(1):316. 10.1186/s12909-020-02193-3 32957972 PMC7504842

