# Supplementary material for: Oncogenetics training in Brazilian medical genetics residency programs: current landscape and challenges
Source: J Community Genet. 2026 Jun 29;17(4):81. doi: 10.1007/s12687-026-00916-5 (PMC13315024; doi:10.1007/s12687-026-00916-5)
Supplement: Supplementary file 1 — Supplementary Material 1 [file 12687_2026_916_MOESM1_ESM.docx]

**SUPPLEMENTARY INFORMATION**

**Oncogenetics Training in Brazilian Medical Genetics Residency Programs: Current Landscape and Challenges**

Amaro Freire de Queiroz Júnior^1^ ([afqjunior@hcpa.edu.br](mailto:afqjunior@hcpa.edu.br))

Angelina Xavier Acosta^2^ ([axacosta@gmail.com](mailto:axacosta@gmail.com))

Carlos Eduardo Steiner^3^ ([steiner@unicamp.br](mailto:steiner@unicamp.br))

Cezar Antônio Abreu de Souza^4^ ([cezarabreu35@gmail.com](mailto:cezarabreu35@gmail.com))

Chong Ae Kim^5^ ([chong.kim@hc.fm.usp.br](mailto:chong.kim@hc.fm.usp.br))

Fernando Regla Vargas^6^ ([fernandorevargas@gmail.com](mailto:fernandorevargas@gmail.com))

Maria Denise Fernandes Carvalho de Andrade^7^ ([dra.denisecarvalho@gmail.com](mailto:dra.denisecarvalho@gmail.com))

Patrícia Santana Correia^8^ ([correia.pat@gmail.com](mailto:correia.pat@gmail.com))

Paulo Ricardo Gazzola Zen^9^ (paulozen[@ufcspa.edu.br](mailto:carlagr@ufcspa.edu.br))

Romina Soledad Heredia^10^ ([romina.rh@gmail.com](mailto:romina.rh@gmail.com))

Victor Evangelista de Faria Ferraz^11,12^ ([vferraz@usp.br](mailto:vferraz@usp.br))

Débora Gusmão Melo^13^ ([dgmelo@unifesp.br](mailto:dgmelo@unifesp.br))*

Patrícia Ashton-Prolla^14,15,16^ ([pprolla@hcpa.edu.br](mailto:pprolla@hcpa.edu.br))*

*(*) should be listed as joint last authors.*

1. Instituto Nacional de Ciência e Tecnologia de Doenças Raras (InRaras) - Hospital de Clínicas de Porto Alegre (HCPA), Porto Alegre, Brazil.

2. Programa de Residência Médica em Genética Médica do Complexo Hospital Universitário Professor Edgar Santos/Maternidade Climério de Oliveira/Universidade Federal da Bahia (Hupes-UFBA), Salvador, Brazil.

3. Programa de Residência Médica em Genética Médica da Universidade Estadual de Campinas (UNICAMP), Campinas, Brazil.

4. Programa de Residência Médica em Genética Médica do Hospital das Clínicas da Universidade Federal de Minas Gerais (UFMG), Belo Horizonte, Brazil.

5. Programa de Residência Médica em Genética Médica do Hospital das Clínicas da Faculdade de Medicina da Universidade de São Paulo (FMUSP), São Paulo, Brazil.

6. Programa de Residência Médica em Genética Médica do Hospital Universitário Gaffrée e Guinle/Universidade Federal do Estado do Rio de Janeiro (UNIRIO) e Instituto Oswaldo Cruz, Fundação Oswaldo Cruz (FIOCRUZ), Rio de Janeiro, Brazil.

7. Programa de Residência Médica em Genética Médica do Hospital Universitário do Ceará/Escola de Saúde Pública do Ceará (ESP/CE), Fortaleza, Brazil.

8. Programa de Residência Médica em Genética Médica do Instituto Nacional de Saúde da Mulher, da Criança e do Adolescente Fernandes Figueira (IFF/Fiocruz), Rio de Janeiro, Brazil.

9. Programa de Residência Médica em Genética Médica da Universidade Federal de Ciências da Saúde de Porto Alegre (UFCSPA)/Irmandade da Santa Casa de Misericórdia de Porto Alegre (ISCMPA), Porto Alegre, Brazil.

10. Programa de Residência Médica em Genética Médica da Escola Superior de Ciências da Saúde (ESCS), Brasília, DF, Brazil.

11. Programa de Residência Médica em Genética Médica do Hospital das Clínicas da Faculdade de Medicina da Universidade de São Paulo (HCFMRP-USP), Ribeirão preto, Brazil.

12. Departamento de Genética da Faculdade de Medicina de Ribeirão Preto da Universidade de São Paulo, Ribeirão Preto, Brazil.

13. Programa de Residência Médica em Genética Médica da Escola Paulista de Medicina, Universidade Federal de São Paulo (UNIFESP), São Paulo, Brazil.

14. Programa de Residência Médica em Genética Médica do Serviço de Genética Médica, Hospital de Clínicas de Porto Alegre (HCPA), Porto Alegre, Brazil.

15. Departamento de Genética, Universidade Federal do Rio Grande do Sul, Porto Alegre, Brazil.

16. Instituto Nacional de Ciência e Tecnologia de Prevenção e Saúde de Precisão em Oncogenética (Prev-Onco), Porto Alegre, Brazil.

**Correspondence to**

Patrícia Ashton-Prolla

Medical Genetics Service

Rua Ramiro Barcelos 2350

Porto Alegre RS 90035-903 - Brazil

Tel: + 55 51 3359-8011

E-mail: [pprolla@hcpa.edu.br](mailto:pprolla@hcpa.edu.br)

**Authors’ ORCID**

Amaro Freire de Queiroz Júnior - 0009-0003-2149-5188

Angelina Xavier Acosta - 0000-0003-1494-1373

Carlos Eduardo Steiner - 0000-0001-5148-3063

Cezar Antônio Abreu de Souza - 0000-0003-4231-1951

Chong Ae Kim - 0000-0002-1754-1300

Fernando Regla Vargas - 0000-0003-1106-4061

Maria Denise Fernandes Carvalho de Andrade - 0000-0003-1890-4721

Patrícia Santana Correia - 0000-0002-5833-6856

Paulo Ricardo Gazzola Zen - 0000-0002-7628-4877

Romina Soledad Heredia - 0009-0005-6588-2643

Victor Evangelista de Faria Ferraz - 0000-0003-0337-4588

Débora Gusmão Melo - 0000-0001-7005-3544

Patrícia Ashton-Prolla - 0000-0002-5093-4739

**Statements and Declarations**

**Author Contribution information**

**Amaro Freire de Queiroz Júnior**: Conceptualization, Methodology, Formal analysis, Data interpretation, Writing – original draft. **Angelina Xavier Acosta**: Data interpretation, Writing – review & editing. **Carlos Eduardo Steiner**: Data interpretation, Writing – review & editing. **Cezar Antônio Abreu de Souza**: Data interpretation, Writing – review & editing. **Chong Ae Kim**: Data interpretation, Writing – review & editing. **Fernando Regla Vargas**: Data interpretation, Writing – review & editing. **Maria Denise Fernandes Carvalho de Andrade**: Data interpretation, Writing – review & editing. **Patrícia Santana Correia**: Data interpretation, Writing – review & editing. **Paulo Ricardo Gazzola Zen**: Data interpretation, Writing – review & editing. **Romina Soledad Heredia**: Data interpretation, Writing – review & editing. **Victor Evangelista de Faria Ferraz**: Data interpretation, Writing – review & editing. **Débora Gusmão Melo**: Conceptualization, Methodology, Formal analysis, Data interpretation, Writing – review & editing, Supervision. **Patrícia Ashton-Prolla**: Conceptualization, Project administration, Methodology, Formal analysis, Data interpretation, Writing – review & editing, Supervision. All authors read and approved the final manuscript.

**Competing Interests**

The authors declare that they have no conflict of interest.

**Evaluation of Oncogenetics Practice in Medical Genetics Residency Programs across Brazil**

You are being invited to participate in a national evaluation regarding the availability of Oncogenetics training within the different Medical Genetics Residency Programs (PRMs) certified by the Ministry of Education (MEC) in Brazil. The objective is to collect information on internship practice and supervision and, by analyzing the results, identify opportunities for improvement in the training of medical geneticists in this field.

This questionnaire has the formal support of the Brazilian Society of Medical Genetics (SBGM).

Responsible for the development of the questionnaire:

Amaro Freire de Queiroz Júnior (Resident Physician - HCPA)

Prof. Débora Gusmão Melo (Medical Geneticist - UNIFESP)

Prof. Patrícia Ashton Prolla (Medical Geneticist - HCPA)

- Identity (your name) of the respondent to this questionnaire:

- Email (your email) of the respondent to this questionnaire:

- What is your role in the Medical Genetics Residency Program (MGRP)?

Mark only one oval.

( ) Coordinator of the MGRP

( ) MGRP Preceptor

( ) Other. Please describe.

- If you selected "Other. Please describe" in the previous item, please describe below.

1. Which institution is the Medical Genetics Residency Program (MGRP) that you are associated with?

2. Is the MGRP service accredited under the Rare Diseases Ordinance (Ordinance No. 199, of January 30, 2014)? If accredited, is it linked as a Specialized Care Service or as a Reference Service?

3. Does the Medical Residency Program offered at your institution include a specific rotation/internship in Oncogenetics?

Mark only one oval.

( ) Yes

( ) No

4. Is the Oncogenetics rotation/internship provided within the institution where the MGRP is based?

Mark only one oval.

( ) Yes

( ) No

5. If you selected "No" in the previous item, please describe below the partner institution(s) that offer(s) the rotation.

6. What is the total hourly workload of the Oncogenetics rotation (practical component), considering case discussion meetings, direct patient care, and resident physician supervision by a preceptor? (in hours across the entire MGRP)

7. Following up on the previous question, what is the total duration, in months, of the Oncogenetics rotation for residents in this MGRP?

8. Following up on the previous question, in which year(s) of the residency does the oncogenetics rotation take place.

Check all that apply.

( ) Year 1

( ) Year 2

( ) Year 3

9. Does the Oncogenetics rotation include a formal theoretical training program?

Mark only one oval.

( ) Yes

( ) No

10. If you selected "Yes" in the previous item, please describe below the number of hours of theoretical activity in the PRM (overall workload, encompassing the entire residency period):

11. Following up on the previous question, in which year(s) of the residency does the theoretical program in oncogenetics take place.

Check all that apply.

( ) Year 1

( ) Year 2

( ) Year 3

12. Is the patient care within the scope of the Oncogenetics rotation characterized by a multidisciplinary approach, involving other professionals in addition to the geneticist, such as psychologists, surgical oncologists, medical oncologists, or other specialists?

Mark only one oval.

( ) Yes

( ) No

13. If you selected "Yes" in the previous item, please describe below the professionals who are part of the multidisciplinary team.

14. Does the multidisciplinary care occur at the same time/appointment slot as the care provided by the medical geneticist, or in a separate slot/time?

Mark only one oval.

( ) Joint care at the same time/appointment slot

( ) Care at a separate time/appointment slot

15. How many oncogenetics patients are seen per week during the rotations (please state the weekly average of patient appointments)?

16. Which professional serves as the preceptor responsible for the Oncogenetics rotation?

Check all that apply.

( ) Medical geneticist

( ) Medical oncologist

( ) Other (please specify)

17. If you selected "Other (please specify)" in the previous item, please describe below.

18. How is the performance of residents evaluated during the oncogenetics rotations, and what evaluation criteria are used?

Check all that apply.

( ) Evaluation by the preceptor

( ) Theoretical exam

( ) Practical exam

19. Following up on the previous question, please mention the evaluation criteria used to analyze the residents' performance.

20. Does your institution, where the PRM is located, participate in the Brazilian Hereditary Cancer Network (ReBraCH)?

Mark only one oval.

( ) Yes

( ) No

21. Are there opportunities for residents to participate in oncogenetics research activities during the MGRP?

Mark only one oval.

( ) Yes

( ) No

22. Is there a possibility of undertaking an internship/rotation at other institutions outside of the institutional program? If so, how many months, and what type of rotation (clinical assistance, laboratory, or research)?

23. What partnerships or collaborations exist between the Oncogenetics PRM and specialized centers, genetics laboratories, or research institutions in the field?

24. Is there specific training on ethical issues related to genetic counseling in oncogenetics?

Mark only one oval.

( ) Yes

( ) No

25. Following up on the previous question, please specify how ethical issues related to genetic counseling are addressed in training — approach via theoretical lecture? Case discussion?
